# 3D-Printed ABS and PLA Scaffolds for Cartilage and Nucleus Pulposus Tissue Regeneration

**DOI:** 10.3390/ijms160715118

**Published:** 2015-07-03

**Authors:** Derek H. Rosenzweig, Eric Carelli, Thomas Steffen, Peter Jarzem, Lisbet Haglund

**Affiliations:** 1 The Orthopedics Research Lab, Department of Surgery, McGill University, Montreal, QC H3G 1A4, Canada; E-Mails: derek.rosenzweig@mail.mcgill.ca (D.H.R.); carelli.eric@gmail.com (E.C.); tsteffen@orl.mcgill.ca (T.S.); 2 McGill Scoliosis & Spine Group, Department of Surgery, McGill University, Montreal, QC H3G 1A4, Canada; E-Mail: pjarzem@gmail.com; 3 McGill University Health Centre, Department of Surgery, Montreal General Hospital, Room C10.148.2, 1650 Cedar Ave, Montreal, QC H3G 1A4, Canada

**Keywords:** 3D printing, chondrocyte, nucleus pulposus, intervertebral disc, PLA, ABS, tissue engineering

## Abstract

Painful degeneration of soft tissues accounts for high socioeconomic costs. Tissue engineering aims to provide biomimetics recapitulating native tissues. Biocompatible thermoplastics for 3D printing can generate high-resolution structures resembling tissue extracellular matrix. Large-pore 3D-printed acrylonitrile butadiene styrene (ABS) and polylactic acid (PLA) scaffolds were compared for cell ingrowth, viability, and tissue generation. Primary articular chondrocytes and nucleus pulposus (NP) cells were cultured on ABS and PLA scaffolds for three weeks. Both cell types proliferated well, showed high viability, and produced ample amounts of proteoglycan and collagen type II on both scaffolds. NP generated more matrix than chondrocytes; however, no difference was observed between scaffold types. Mechanical testing revealed sustained scaffold stability. This study demonstrates that chondrocytes and NP cells can proliferate on both ABS and PLA scaffolds printed with a simplistic, inexpensive desktop 3D printer. Moreover, NP cells produced more proteoglycan than chondrocytes, irrespective of thermoplastic type, indicating that cells maintain individual phenotype over the three-week culture period. Future scaffold designs covering larger pore sizes and better mimicking native tissue structure combined with more flexible or resorbable materials may provide implantable constructs with the proper structure, function, and cellularity necessary for potential cartilage and disc tissue repair *in vivo.*

## 1. Introduction

Advancements in scaffold design combined with rapidly advancing techniques in additive manufacturing and availability of high-resolution desktop 3D printers are allowing fabrication of biodegradable biomimetics with suitable form and function for tissue engineering and organ replacement applications. Additive manufacturing refers to the process of creating solid objects through the deposition of sequential layers, usually digitally designed with 3D computer-aided design software [1]. Due to the cost-effectiveness, the type of additive manufacturing commonly used today is fused filament fabrication [2] or fused deposition modeling [3,4]. These identical processes use heated extruders to melt plastic and build the layers of the designed scaffold [1]. Biocompatible materials commonly used for implantable constructs include polylactic acid (PLA), polyglycolic acid (PGLA), polycaprolactone (PCL), and blends of polyethylene glycol (PEG). However, the most widely used materials in desktop 3D printers are PLA and acrylonitrile butadiene styrene (ABS), the latter being non-biodegradable and generally non-biocompatible with the exception of ABS M30i plastics, which are suitable for biotechnology applications. PLA and PLA-polymer blends in combination with various hydrogels (composite scaffolds) have been shown to be effective scaffold agents for cartilage [5,6,7], intervertebral disc [7,8,9], and bone [6,10,11] tissue engineering. The use of 3D printing for such material scaffolds allows for highly reproducible and uniform open-pore size and potential orthogonal geometry, which can be tailored to mimic the tissue composition of choice.

Articular cartilage and intervertebral disc (IVD) degeneration directly contribute to joint and back pain, affecting multitudes of people and direct and indirect healthcare costs worldwide [12,13]. Both of these soft tissues are rich in collagen (tensile strength) and proteoglycan, the latter of which holds water and provides the tissues with the main function to absorb and distribute a mechanical load. The resident cells of hyaline cartilage are chondrocytes, while IVDs consist of two distinct regions with resident cells: the central gelatinous nucleus pulposus (NP) and outer fibrocartilaginous annulus fibrosus (AF). Both hyaline chondrocytes and IVD cells are responsible for matrix remodeling and the same cell is responsible for both synthesis and breakdown [14,15]. Physiological loading of these tissues is essential for optimal cell function in matrix maintenance [16,17]. However, disruption of matrix maintenance occurs when injurious compressions or mechanical overloads to these tissues occur (see our new paper).

Both articular cartilage and IVDs are avascular tissues with limited self-repair capacity. Trauma or injury to the tissue disrupts matrix composition and function. Slowly changing biomechanical properties post-injury are thought to be the initiating factor of increased inflammatory and catabolic processes, which result in osteoarthritis and degenerative disc disease [18,19,20,21]. To create a suitable three-dimensional scaffold and circumvent progression of degenerative disease, additive manufacturing has been proposed to mimic tissue shape and mechanical properties of acutely injured tissue [22,23]. Cell implantation strategies and artificial tissue replacements have had limited success in repairing these damaged tissues, and often end-stage invasive surgical procedures are required to alleviate inflammatory and mechanical pain. To this end, scaffold designs integrating mechanically competent biodegradable synthetic and biologically generated materials (*i.e.*, composite polymers coated with collagen hydrogels) are showing promise within the field of cartilage and disc tissue engineering and regenerative medicine [24,25,26,27].

Generation of useful polymeric scaffolds for tissue engineering must account for several parameters including scaffold degradation rates, biocompatibility, host tissue integration, mechanical stability, and even optimal pore size. Several studies have suggested the importance of scaffold pore size in tissue engineering. For bone tissue engineering, the critical pore size of composite scaffolds is about 100 µm [28,29], while optimal pore size for bone growth is about 300–350 µm [30]. Furthermore, even a larger polymer scaffold pore size (>500 µm) is conducive towards soft tissue ingrowth [31,32]. In addition to pore size, several other parameters must be taken into account as well when generating such scaffolds. Scaffold porosity, surface area, protein coating, mechanical properties and diffusion, and fluid flow rates all may affect cell seeding, adherence, and growth. The line and layer resolution for the printer also may be a limitation of all of the abovementioned parameters since extruder nozzles typically have openings between 0.25 and 0.5 mm. We hypothesize here that both chondrocytes and NP cells will dedifferentiate and produce small quantities of proteoglycan when cultured on large-pore scaffolds. We also test whether cells will grow and deposit matrix more readily on PLA *versus* ABS scaffolds. 3D-printed scaffolds allow for rapid and favorable architecture design for optimizing cell growth and matrix production, and they provide valuable information towards better scaffold design for cartilage and disc repair or replacement.

## 2. Results

### 2.1. ABS and PLA Scaffold Design and Properties

**Figure 1:**
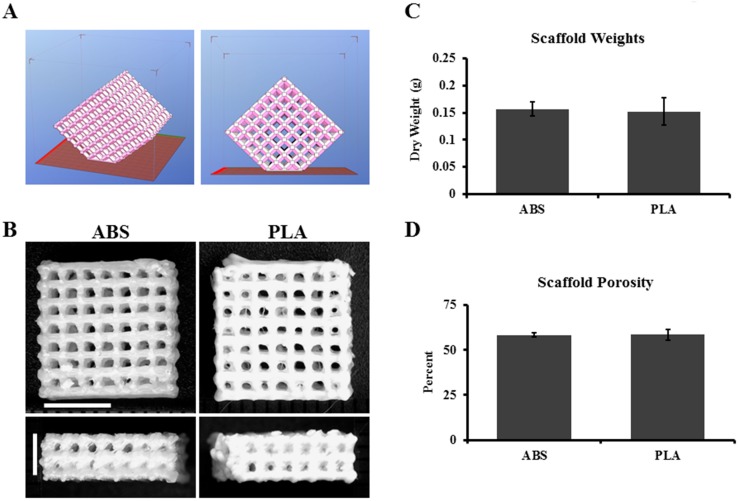
ABS and PLA scaffold design. Construct design was downloaded from the freeware website http://www.thingiverse.com/thing:45228/#files. (**A**) Representative *.stl* images of scaffold structure; (**B**) representative images of printed ABS and PLA scaffolds. Horizontal scale bar: 0.5 cm; vertical scale bar: 0.3 cm; (**C**) Quantification of scaffold weight; (**D**) quantified porosity of the ABS and PLA scaffolds used in the studies. Error bars represent ± SD, *n* = 6.

In order to determine the feasibility of using large-pore 3D-printed ABS and PLA thermoplastics for both cartilage and NP tissue engineering applications, 3D scaffolds were designed and printed as described in the Methods section (Figure 1A,B). The average weights for ABS and PLA scaffolds were determined to be quite similar (0.158 ± 0.013 and 0.152 ± 0.025 g, respectively) (Figure 1C). The theoretical porosity of scaffolds was calculated to be 48% for both scaffolds, and measured porosity was found to be 58.3% ± 1.06% for ABS and 58.5% ± 3.01% for PLA (Figure 1D). The fused deposition model printed spacing of ~700 µm pore size theoretically provides ample room for cell ingrowth and neo-matrix production.

**Figure 2:**
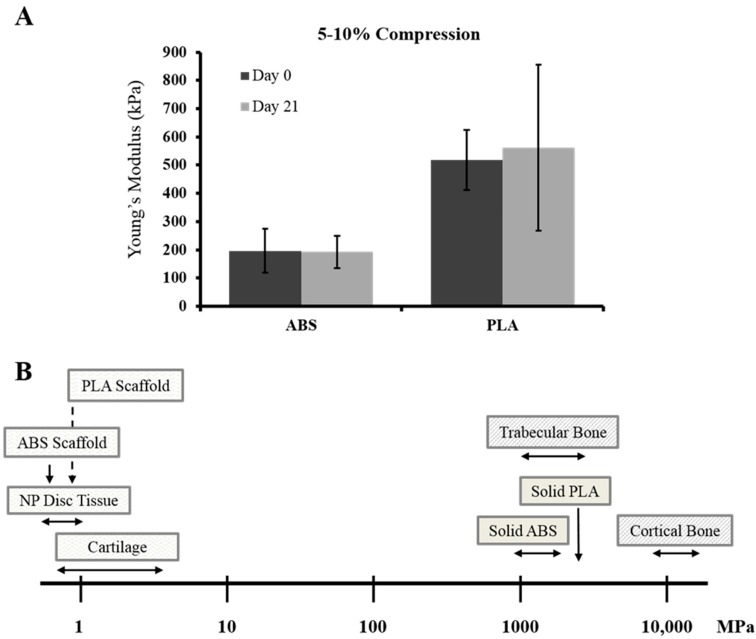
Mechanical testing of ABS and PLA scaffolds. (**A**) Young’s modulus was calculated between 5%–10% compressive strain/strain curves for freshly printed (day 0) and culture medium bathed (day 21) ABS and PLA scaffolds. For each set, *n* = 5. Error bars represent ± SD; (**B**) Cartoon representation of mechanical test data for printed scaffolds placed in context of known mechanical bulk properties of ABS and PLA plastics, as well as human NP tissue, articular cartilage, and bone. Horizontal arrows indicate a range of stiffness, while downward arrows indicate exact stiffness.

Implantable biomaterials such as PLA are known to degrade over time. To determine the mechanical strength at time of printing or after 21 days in culture media, scaffold compressive properties were measured. Between physiological strains of 5%–10%, no significant differences were observed in stiffness between freshly printed (day 0) or 21 days-cultured ABS (~197 and ~193 kPa) or PLA (~520 and ~560 kPa) scaffolds (Figure 2A). We placed these printed scaffolds in the context of known compressive properties of solid ABS and PLA, or those of human articular cartilage (Figure 2B). The printed scaffolds are significantly less stiff than solid plastics, and surprisingly show compressive properties comparable to native tissue when strained between 5% and 10%.

### 2.2. Chondrocytes and NP Cells Attach to and Populate the Pores of ABS and PLA Scaffolds

**Figure 3:**
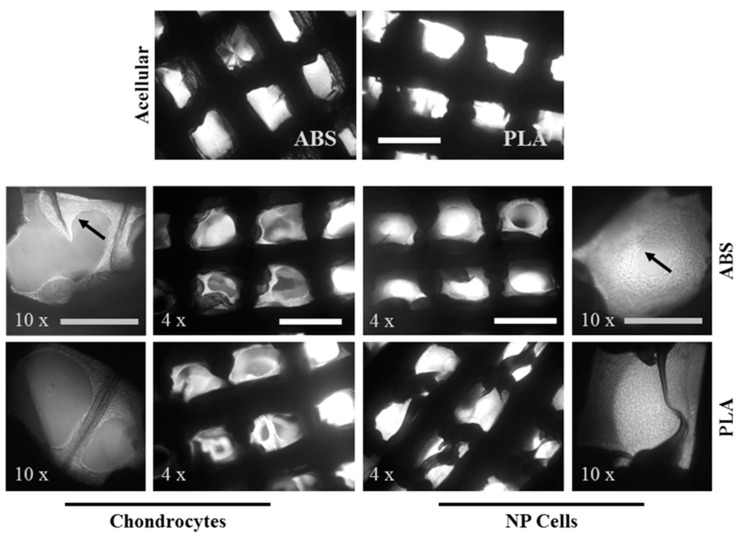
Cell ingrowth on ABS and PLA scaffolds. Representative phase contrast images of cell-seeded scaffolds at 4× and 10× magnification after 21 days of culture (*n* = 4). Black arrows indicate cell growth and neo-tissue deposition. White scale bar represents 1 mm; grey scale bar represents 500 µm.

Scaffolds were seeded with either primary chondrocytes or primary NP cell and cultured for 21 days. Cell adhesion and growth were monitored at 7, 14, and 21 days with phase contrast imaging. Adhesion of cells was immediately visible, and growth of both cell types was increasing at 7 and 14 days (data not shown). Figure 2 shows that by 21 days of culture, chondrocytes have grown over most of the surface of both ABS and PLA scaffolds, with some in-growth into the open pores. NP cells, however, almost completely fill the open pores via growth and neo-matrix production (Figure 3).

**Figure 4:**
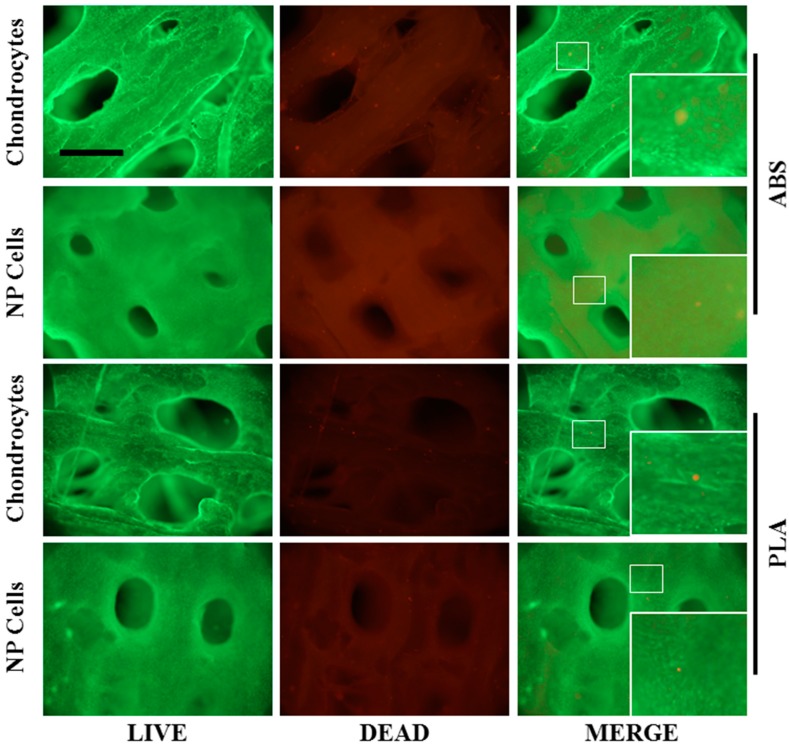
Cell viability on ABS and PLA scaffolds. Live/dead assay was performed on chondrocytes and NP cell-seeded ABS and PLA scaffolds after 21 days’ culture. Live cells are shown in green (calcein AM) while dead cells are shown in red (ethidium homodimer). Representative images (*n* = 4) show that the number of dead cells on either scaffold is negligible. Inset panels are enlarged 3× from indicated regions, and show individual live and dead cells. Scale bar: 1.0 mm.

To account for chondrocyte and NP cell viability on seeded ABS and PLA scaffolds, LIVE/DEAD assays were performed after 21 days in culture. Both chondrocytes and NP cells showed very high viability on ABS and PLA scaffolds (Figure 4). Both chondrocytes and NP cells surrounded and filled in the scaffolds with dense layers of cells and matrix-like tissue on both ABS and PLA scaffolds. Chondrocyte growth appeared less dense than PLA, yet in-growth to the pore spaces was somewhat evident.

### 2.3. Chondrocytes and NP Cells Produce Proteolgylcan on ABS and PLA Scaffolds

**Figure 5:**
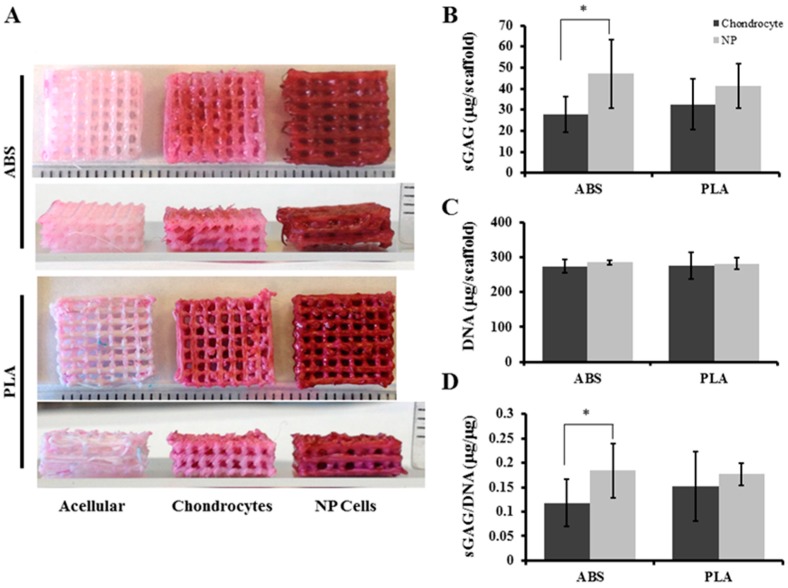
Proteoglycan analysis of scaffolds. (**A**) Fixed acellular, chondrocyte-, and NP-seeded ABS and PLA scaffolds were stained with safranin-O/fast green after 21 days’ culture. Representative images (*n* = 4) showing that both chondrocyte- and NP-seeded ABS and PLA scaffolds retained intense safranin-O staining. Ruler ticks represent millimeters; (**B**) DMMB assay for quantifying sulfated GAG in total extract per scaffold (in 1 mL); (**C**) DNA content was quantified by HOECHST 33258 assay per scaffold; (**D**) The sGAG/DNA ratio was determined. Error bars represent ± SD. * indicates *p* < 0.05.

Histological staining was performed to assess the distribution of proteoglycan and glycosaminoglycan (GAG) production on and within ABS and PLA constructs. After 21 days’ culture, chondrocytes showed moderate safranin-O staining evenly distributed on ABS and PLA scaffolds, compared to acellular controls (Figure 5A). NP cells showed more intense safranin-O staining on both ABS and PLA scaffolds compared to chondrocyte-seeded scaffolds and acellular controls, indicating high production of proteoglycans (Figure 5A). After 21 days’ culture, total sulfated-GAG from chondrocyte-seeded ABS scaffolds was 27.8 ± 8.6 µg/mL. NP-seeded ABS scaffolds contained 46.9 ± 16.2 µg/mL sulfated-GAG, which was significantly (*p* = 0.026) more than the chondrocytes (Figure 5B). Chondrocyte-seeded PLA scaffolds contained 32.6 ± 12.3 µg/mL sulfated-GAG, whereas NP-seeded PLA scaffolds contained 41.3 ± 10.5 µg/mL sulfated-GAG (*p* = 0.139) (Figure 5B).

Cell populations expanded over the 21 days of culture, and DNA content for the cell-seeded scaffolds was measured, showing similar growth of chondrocytes and NP cells on both scaffold types. Chondrocyte-seeded ABS scaffolds contained 273.67 ± 19.5 µg DNA, while NP-seeded ABS scaffolds contained 275.0 ± 6.56 µg DNA (Figure 5C). Chondrocyte-seeded PLA scaffolds contained 284.67 ± 38.84 µg DNA, and NP-seeded PLA scaffolds contained 282 ± 17.86 µg DNA (Figure 5C). No differences in population doublings for either chondrocytes or NP cells on ABS or PLA were observed (data not shown). To account for cell populations towards proteoglycan production, extracted GAG was normalized to total DNA content in extracts. Chondrocyte-seeded ABS scaffolds contained 0.11 ± 0.048 µg GAG per µg DNA while NP-seeded ABS scaffolds contained significantly more (0.183 ± 0.07 µg GAG per µg DNA; *p* = 0.02) (Figure 5D). No significant difference in proteoglycan content was found between chondrocyte-seeded and NP-seeded PLA scaffolds (0.152 ± 0.055 and 0.177 ± 0.023 µg GAG per µg DNA, respectively; *p* = 0.222) (Figure 5D).

### 2.4. Chondrocytes and NP Cells Produce Collagen Type II on ABS and PLA Scaffolds

**Figure 6:**
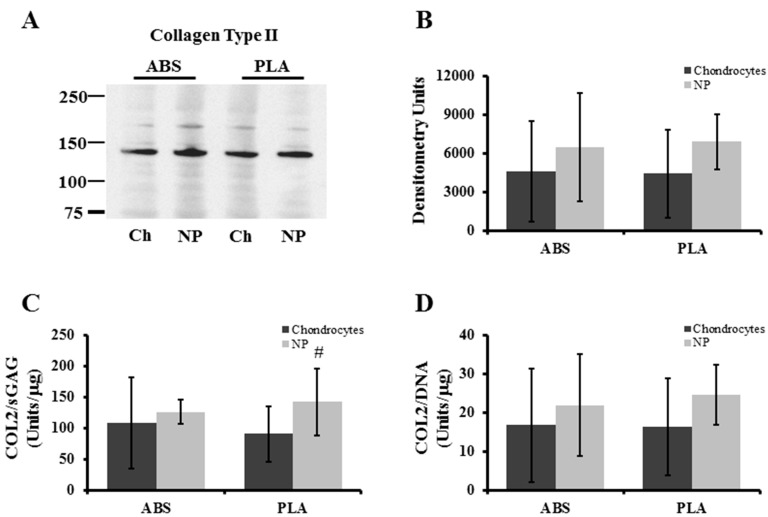
Collagen type II analysis of cell-seeded scaffolds. (**A**) Representative Western blot probing extracts for newly synthesized, non-crosslinked collagen type II; (**B**) densitometry quantification for collagen type II deposition on scaffolds; (**C**) extractable collagen type II band density/sGAG was determined; (**D**) extractable collagen type II band density/DNA was determined. Error bars represent ± SD. # indicates *p* = 0.0601.

Western blot analysis of protein extracts from chondrocyte and NP-seeded ABS and PLA scaffolds (21 days culture) were performed (Figure 6A). Densitometry analysis showed that NP cells produced slightly, but insignificantly, more extractable (non-crosslinked) collagen type II protein (Figure 6B). The ratio of collage type II to proteoglycan production and total cell population was also taken into account for the cell-seeded scaffolds. Chondrocyte-seeded ABS scaffolds contained 108.11 ± 73.26 densitometry units per µg sGAG, while NP-seeded ABS scaffolds contained 126.14 ± 19.92 densitometry units per µg sGAG (*p* = 0.291) (Figure 6C). Chondrocyte-seeded PLA scaffolds contained 90.86 ± 44.6 densitometry units per µg GAG, while NP-seeded PLA scaffolds contained a higher collagen II to GAG ratio approaching statistical significance (142.15 ± 53.58 densitometry units per µg sGAG; *p* = 0.061) (Figure 6C). When measured against the total DNA, chondrocyte-seeded ABS scaffolds contained 16.72 ± 14.3 densitometry units per µg DNA while NP-seeded ABS scaffolds contained 21.94 ± 13.12 densitometry units per µg DNA (*p* = 0.269) (Figure 6D). Chondrocyte-seeded PLA scaffolds contained 16.29 ± 12.61 densitometry units per µg DNA while NP-seeded PLA scaffolds contained a slightly higher collagen II to proteoglycan ratio (24.59 ± 7.79 densitometry units per µg DNA; *p* = 0.109) (Figure 6D).

### 2.5. Chondrocytes and NP Evenly Distribute Collagen II and Aggrecan on ABS and PLA Scaffolds

**Figure 7:**
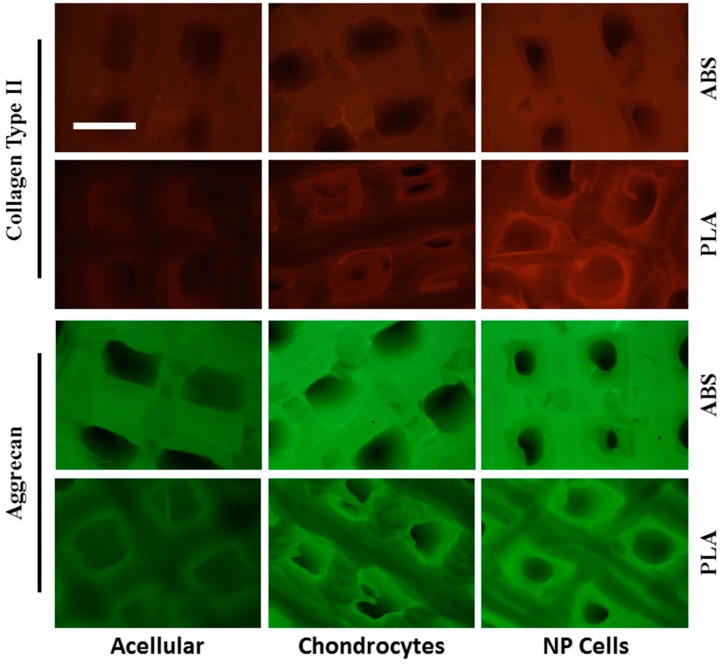
Immunofluorescence analysis of cell-seeded scaffolds. Acellular, chondrocyte-, and NP-seeded ABS and PLA scaffolds were probed by immunofluorescence for collagen type II (red) and aggrecan core protein (green) after 21 days’ culture. Acellular scaffolds show some autofluorescent background. Cell-seeded scaffolds produce fluorescent signals well above background, specifically in regions within the pores, where only cells are growing. Scale bar: 1.0 mm.

Distribution of collagen type II and aggrecan core protein on cell-seeded ABS and PLA scaffolds was also assessed by immunofluorescence. Both scaffold types displayed some background auto-fluorescence under the two wavelengths used. After 21 days’ culture, chondrocytes cultured on ABS and PLA scaffolds showed specific fluorescent signals well above acellular background levels for both collagen type II (red signal) and aggrecan core protein (green signal) (Figure 7). Likewise, NP cells cultured on both ABS and PLA scaffolds showed signals well above acellular controls for both collagen type II and aggrecan (Figure 7). Taken together, these immunofluorescence data show even distribution of matrix deposition on the scaffolds and further support western blot and DMMB analysis of protein extracts.

## 3. Discussion

The quickly advancing development of next-generation desktop 3D printers with high-resolution and multiple print materials is increasing the speed at which novel ideas for tissue engineering, regenerative medicine, and whole organ replacement can be prototyped and assessed. To explore the hypothesis that chondrocytes and NP cells will dedifferentiate when cultured on large-pore 3D-printed scaffolds and whether cells will grow better on PLA *versus* ABS, ~700 µm pore-sized orthogonal ABS and PLA scaffolds were seeded with the primary, unpassaged cells and cultured for 21 days. Primary bovine chondrocyte and NP cells display high viability, adhere well, and proliferate readily on either construct type. Chondrocytes and NP cells proliferated and filled in the empty pore spaces, by generating neo-matrix consisting of both aggrecan proteoglycan and collagen type II protein after three weeks in culture. NP cells generated more proteoglycan than chondrocytes, but no difference was observed between the ABS and PLA scaffolds. Mechanical testing of the ABS and PLA scaffolds suggested that after 21 days in a culture medium, their properties remain unchanged. Together, these data lay an important foundation for designing and generating 3D-printed constructs that contain mechanical and structural properties more closely resembling native tissues. These 3D-printed scaffolds also provide a suitable environment for cell growth and matrix regeneration for cartilage and disc repair applications.

Additive manufacturing and rapid prototyping are increasingly changing the landscape of tissue engineering for cartilage and disc repair and regeneration. Three-dimensionally printed PLA scaffolds have already been used with some success in studies on cartilage and IVD repair [8,9,33]. Similar 3D-printed thermoplastics, such as polycaprolactone, have also showed some success in tissue engineering of cartilage and discs [34,35,36,37], even producing constructs mimicking the human ear for plastic surgery applications [38] and a whole trachea to resolve breathing problems [39,40]. At the very least, 3D printing with these materials combined with primary cell seeding is a gateway to more appropriate tissue engineering strategies.

A major goal in cartilage and IVD tissue engineering is to produce neo-tissues that resemble native tissue both structurally and functionally. It is desirable for the engineered tissue to contain ample proteoglycan (aggrecan and sulfated GAGs) as well as ample collagen type II (for cartilage and NP tissue), all while maintaining mechanical stability. This will directly influence the function of the neo-tissue generated. Studies on the chondrogenesis of PLA or PLA hybrid scaffolds have shown increased proteoglycan and collagen type II production [5,41]. These same biochemical markers have been shown to be present in PLA scaffolds for IVD tissue engineering [8,9]. In both cases, simplistic scaffold architecture was not tested as a variable influencing matrix production. The present study shows that simple uniform ~700 µm pore size PLA and ABS scaffolds are suitable for chondrocyte and NP cell growth and collagen type II and proteoglycan production. Our qualitative data analysis, however, indicated a lower GAG/collagen ratio for both chondrocytes and NP cells compared to values in native tissues [42]. This may be the result of the static culture of these cell-seeded scaffolds, since mechanical loading in the physiological range is known to significantly enhance proteoglycan production in cartilage and NP tissue [19,43]. It is important to note that in the present study, NP cells produced more proteoglycan on both scaffolds compared to chondrocytes. This indicates that the cells do not dedifferentiate and instead maintain their phenotype since NP cells are known to produce more proteoglycan than chondrocytes *in vivo* [42]. We also demonstrate here that culturing cells on ABS scaffolds is comparable to culturing cells on the more widely used biocompatible PLA scaffolds. Newer and more biocompatible ABS polymers are emerging, such as ABS M30i, indicating the potential for future use in tissue engineering constructs.

Another goal in cartilage and IVD tissue engineering is to produce neo-tissues that function in a biomechanically similar way to native tissues. Collagen, hyaluronic acid, or chitosan hydrogels provide a suitable environment for chondrocyte and IVD cell growth and matrix deposition; however, the mechanical properties of the engineered tissues are often not strong enough to endure physiological loading upon clinical implantation. Ideally, cartilage constructs should display compressive stiffness in the 0.5–6.0 MPa range (depending on physical activity) [44,45,46], while nucleus pulposus (central gelatinous portion of IVD) constructs should withstand loads ranging from 0.01–1.5 MPa [47,48]. Several studies on engineered cartilage constructs report mechanical properties in the kilopascal range, not nearly strong enough for physiological loading. Indeed, we have demonstrated promising biochemical properties for cartilage/collagen constructs, yet the mechanical properties were not ideal to withstand high compressive loads [49].

One advantage to using 3D-printed scaffolds is that they inherently contain mechanical properties comparable to or stronger than the native tissues. In fact, the compressive properties of the scaffolds used in this study are still too stiff for use in tissue repair. Since the seeded cells are able to produce ample biological matrix, future scaffold designs should contain more optimized geometry with perhaps thinner support struts, thereby rendering mechanical properties closer to that of native tissues. The present study shows the extent to which cells seeded on large-pore constructs can produce the biochemical components while maintaining strong mechanical properties. Even after the scaffolds biodegrade at some later time point, this theoretically will allow such engineered tissues to withstand high loads immediately after implantation.

## 4. Experimental Section

### 4.1. 3D-Printed Constructs

ABS and PLA filament (1.75 mm) were purchased from MakerBot (New York City, NY, USA). A 3D, uniform, open-pore orthogonal “matrix” construct *.stl* file was downloaded from the freeware website (http://www.thingiverse.com/thing:45228/#files) and modified for use with an Afinia H-series H479 desktop 3D printer. All ABS and PLA “matrix” constructs were printed with perpendicular 10 mm lines with 0.7 mm thickness. On the Afinia printer, ABS plastic extrudes at a fixed temperature of 260 °C, while PLA plastic extrudes at a fixed temperature of 210 °C. For all prints, speed control was set to “fine,” and the bed temperature was set to 105 °C. Total final dimensions were 1 × 1 × 0.3 cm. (Figure 1). To promote cell adhesion, all printed grid constructs were first soaked in 70% ethanol overnight, washed with sterile PBS, and coated with 50 µg/mL collagen type I (Carlsbad, CA, USA) and 0.1% Poly-l-Lysine (Sigma, Burlington, ON, Canada) for 2 h at 37 °C. After scaffold surface treatment, constructs were washed three times with sterile PBS.

### 4.2. Porosity of 3D-Printed Scaffolds

Theoretical calculation of scaffold porosity was performed by dividing the theoretical void-space (volume of open pores) divided by the total volume of the scaffolds. This was based on the 3D orthogonal model, which set pore size to 0.7 × 0.7 × 10 mm dimensions in both the *x* and *y* plane, while there was 0.7 × 0.7 × 3.5 mm in the *z* plane. Actual porosity was calculated by Archimedes’ principle. Dry-weights and void volumes were determined for sealed ABS and PLA (*n* = 5 for each). Void-weight, divided by total weight and multiplied by 100, gave the actual measured porosity.

### 4.3. Chondrocyte and NP Cell Isolation

Primary bovine NP cells were isolated from dissected nucleus pulposus from caudal discs, and primary bovine chondrocytes were isolated as described previously [50,51] and obtained from a local slaughterhouse. Approximately 5 g of NP and cartilage tissue were washed in sterile phosphate buffered saline (PBS) supplemented with antibiotics and cut into 1–2 mm pieces. The tissue was digested separately overnight in a T-75 flask containing 30 mL of growth medium (high-glucose DMEM; 0.1 mM Nonessential Amino Acids; 10 mM HEPES; 1 mM sodium pyruvate; 50 µg/mL ascorbic acid, 10% fetal bovine serum; and 1% penicillin-streptomycin-glycine solution [10,000 units/mL of penicillin, 10,000 µg/mL of streptomycin]) supplemented with 1.5 mg/mL collagenase type II (Invitrogen/Gibco, Burlington, ON, Canada). The digest was passed through a 100 µm filter (BD Biosciences, Mississauga, ON, Canada) and centrifuged at 300× *g* for 5 min. Pelleted chondrocytes and NP cells were washed with sterile PBS and centrifuged again at 300× *g* for 5 min. Cells were resuspended in 10 mL of growth medium and counted. For initial assessment of effects of ABS and PLA on cell viability, 100,000 chondrocytes or NP cells were plated in six-well plates in the presence of 3, 5, and 7 mm 3D-printed circular wafers. Five ABS and PLA constructs per donor animal (*n* = 4) were placed in 24-well plates and 500,000 of each cell type was seeded. Five of each construct were seeded so as to have enough constructs to analyze viability, total protein and proteoglycan content, histological analysis, and collagen type II and aggrecan immunofluorescence detection. The medium was changed every two days throughout experiments.

### 4.4. Cell Attachment, Growth, and Viability

Monolayer cell culture in the presence of ABS and PLA circular wafers was monitored over seven days, while cell adhesion and overgrowth on constructs was monitored every three days. Phase contrast images for both monolayer and construct cultures were acquired on a Zeiss Axiovert 40C microscope equipped with a Canon Powershot A640 digital camera attached to a Zeiss MC80DX 1.0× tube adapter. Cell viability in the presence of ABS and PLA circular wafers (seven days’ monolayer culture) or on the ABS and PLA constructs (21 days’ 3D culture) was determined by LIVE/DEAD assay prepared in serum-free Dulbecco’s Modified Eagle Medium according to the manufacturer’s instructions. Images were captured and viability was quantified from three random positions from four independent experiments using an Olympus IX81 inverted fluorescence microscope. All images were captured using both 10× (monolayer cultures) and 4× (constructs) objectives with MAG Biosystems Software 7.5 (Photometrics, Tucson, AZ, USA).

### 4.5. Histological and Immunofluorescence Analysis

After 21 days in culture, ABS and PLA constructs (acellular, chondrocyte-seeded, and NP-seeded) were fixed in 4% paraformaldehyde. Once complete set (*n* = 4) was stained with Fast-Green for 5 min, and then washed with water for 10 min. After a brief rinse with 1% acetic acid, all samples were stained with Safranin-O for 5 min, followed by extensive washing with water. Images were captured with a Canon Powershot A640 digital camera. An additional two sets of fixed ABS and PLA constructs (acellular, chondrocyte-seeded, and NP-seeded) were treated with permeabilization buffer (PBS, 1% BSA, 0.1% Triton-X100) for 45 min. Permeabilized samples were then incubated with antibodies against collagen type II (1:100, Abcam, Cambridge, MA, USA) and aggrecan (1:200, Developmental Studies Hybridoma Bank, University of Iowa, Iowa City, IA, USA) overnight at 4 °C. Samples were washed three times in PBS and then incubated with either Alexa Fluor 488 Goat anti-Mouse IgG (1:250, Invitrogen) or Alexa Fluor 568 Goat anti-Rabbit IgG (1:250, Invitrogen) for 1.5 h at room temperature. Samples were washed and mounted with Fluoroshield with DAPI (Sigma) and visualized on an Olympus IX81 inverted fluorescence microscope.

### 4.6. Western Blot

Proteins were extracted from scaffolds (with very similar weights) for 48 h at 4 °C on a rocker in 1 mL of 4 M guanidine hydrochloride (GuHCl), 50 mM sodium acetate, pH 5.8, 10 mM EDTA, and protease inhibitors [52]. Twenty microliter aliquots of protein extract (described above) were precipitated using nine volumes of 100% ethanol. Precipitates were recovered by centrifugation (30 min at 4 °C). Pellets were washed once each with 75% ethanol and 95% ethanol before being dried and redissolved in 20 µL of sample loading buffer, and placed at 65 °C for 5 min. Ten microliters of each sample were subjected to 4%–12% gradient SDS-PAGE gel electrophoresis and then transferred to nitrocellulose membranes. Membranes were blocked in 5% BSA for 45 min and probed with rabbit polyclonal antibodies against collagen type II (1:1000; Abcam 1 mg/mL, ab34712). Membranes were washed three times in TBST followed by incubation with anti-rabbit HRP-conjugated secondary antibody (1:5000, Santa Cruz Biotechnology, Dallas, TX, USA) for 45 min. Membranes were then washed three times in TBST for 10 min, and developed using Western Lightning Plus-ECL (Perkin Elmer, Waltham, MA, USA) and an Image Quant LAS 4000 (GE Healthcare Bio-Sciences, Baie d’Urfe, QC, Canada).

### 4.7. Proteoglycan Quantification

Sulfated glycosaminoglycans (GAGs) were quantified from guanidine HCl extracts by a modified dimethyl methylene blue (DMMB) assay [53]. Samples were diluted to fall in the middle of the linear range of the standard curve. In order to adjust for any interference by GuHCl, an equivalent amount of 4 M GuHCl was added to standards. Samples were loaded in Costar Easy-wash 96-well plates and absorbance was determined using a Tecan Infinite M200 PRO (Tecan, Männedorf, Switzerland) and analyzed with i-control 1.9 software (Tecan).

### 4.8. Mechanical Testing

Exact ABS and PLA scaffold heights were measured using Mitutoyo^®^ calipers. A load-to-failure protocol applied axial compression scaffold surfaces at a rate of 0.1 mm/s until heights were reduced by 40% (approximately 1.2 mm) using a Mini Bionix 858 MTS machine. Displacement (mm), load (N), and time to failure (s) data were collected at a sampling rate of 100 Hz using the built-in data acquisition boards and MTS software Teststar II (MTS Systems, Eden Prairie, MN, USA). Young’s modulus was determined from the slope of stress-strain curves between 5%–10%. As a control, freshly printed scaffolds or scaffolds bathed in growth medium (with media changes every three days) for 21 days were tested.

### 4.9. Statistical Analysis

All statistical analyses were performed comparing NP cells to chondrocytes either on ABS or PLA scaffolds using paired *t*-tests from at least four independent experiments using four individual animal donors. All *p* values less than 0.05 were considered to be statistically significant differences. Analyses were performed using Graphpad Prism 6.0 (Graphpad Software, La Jolla, CA, USA).

## 5. Conclusions

The rapid rise of additive manufacturing by way of desk-top 3D printers and the availability of multiple functional materials to use in such systems are paving the way for faster and improved construct design for tissue engineering applications. ABS and PLA scaffolds provide mechanical stability for tissue engineering. We show that cells grow well and produce ample matrix within large-pore (~700 µm) scaffolds, presenting key components to cartilage and IVD tissue engineering. Moreover, NP cells produce more proteoglycan than chondrocytes, while both cell types produce similar amounts of collagen type II (regardless of scaffold type) indicating proper cell phenotype. Design of new scaffold architectures novel materials with more elastic properties should therefore be considered for improved mechanical function and enhanced matrix production in applications of cartilage and IVD tissue regeneration.
